# Supplementary material for: Analysis of PPARγ Signaling Activity in Psoriasis
Source: Int J Mol Sci. 2021 Aug 10;22(16):8603. doi: 10.3390/ijms22168603 (PMC8395241; doi:10.3390/ijms22168603)
Supplement: Supplementary file 1 [file ijms-22-08603-s001.zip › Supplemental materials_Analysis of PPARg signaling activity in psoriasis/Pathway models/Models images and html files/PPARG inhibits phenotypes assotiated with psoriasis/134932.html]

body weight loss


# Disease body weight loss

|  |  |
| --- | --- |
| URN | urn:agi-meshdis:Weight%20Loss |
| Total Entities | 2 |
| Connectivity | 6182 |
| Name | body weight loss |

---

|  |  |
| --- | --- |
| ChildConcepts | neonatal weight loss |
|  | emaciation |

---

|  |  |
| --- | --- |
| Pathway | PPARG inhibits phenotypes assotiated with psoriasis |
|  | efects selected |

---

|  |  |
| --- | --- |
| MedScan ID | 9004397 |

---

|  |  |
| --- | --- |
| Alias | Weight Loss Finding |
|  | body weight losses |
|  | Loss of Weight |
|  | Weight Losses |
|  | Progressive Weight Loss |
|  | progressive loss of weight |
|  | decrease in weight |
|  | decreased in weight |
|  | Weight Decrease |
|  | Abnormal Loss of Weight |
|  | Body Weight Decreases |
|  | Weightloss |
|  | decreases in body weight |
|  | Weight Decreased |
|  | Weight Reductions |
|  | Weight Decreasing |
|  | Weight Reduction |
|  | Weight Loss |
|  | Body Weight Decrease |
|  | decreases in weight |
|  | Decrease in Body Weight |
|  | Weight Decreases |
|  | Weight Loss Findings |
|  | body weight loss |

---

|  |  |
| --- | --- |
| MeSH ID | D015431 |
|  | M0023779 |

---

|  |  |
| --- | --- |
| MeSH Heading | Weight Loss |

---
